# Implementing a Mixed Health Service Model as an Informed Modality to Enhance Prevention and Promote Workplace Health in the Greek Regional Public Sector: A Pilot Study in Crete

**DOI:** 10.3390/healthcare13182337

**Published:** 2025-09-17

**Authors:** Nikos Frantzeskakis, Maria Tziraki, Marios Spanakis, Spyridoula D. Katsarou, Nikolaos Papadopoulos, Manolis Linardakis, Charikleia Vova-Chatzi, Apostolos Kamekis, George Pitsoulis, Antonios Papadakis, Emmanouil K. Symvoulakis

**Affiliations:** 1 Locum of Preventive Care and Workplace Health, Region of Crete, 71201 Heraklion, Greece; med4p1030424@med.uoc.gr (N.F.); marisatziraki@gmail.com (M.T.); spyridoulakats.30@gmail.com (S.D.K.); apostoloskamekis@gmail.com (A.K.); gpitsoulis@crete.gov.gr (G.P.); apapadakis@crete.gov.gr (A.P.); 2 Department of Social Medicine, School of Medicine, University of Crete, 70013 Heraklion, Greece; linman@uoc.gr; 3 Clinic of Social and Family Medicine, School of Medicine, University of Crete, 70013 Heraklion, Greece; medp2012014@med.uoc.gr (N.P.); xaravova@gmail.com (C.V.-C.); 4 Public Health Authority of the Region of Crete, 71201 Heraklion, Greece; 5 Region of Crete, 71201 Heraklion, Greece

**Keywords:** preventive healthcare, proactive health, workplace health, occupational health, integrated care, primary care, vaccination coverage, metabolic risk factors, stress assessment, public health intervention, social medicine

## Abstract

**Background/Objectives**: Preventive care in Greece remains underdeveloped, especially in workplace settings. To address this, a mixed service model was piloted to integrate preventive and occupational health for public sector employees in Region of Crete. **Methods**: Between January and July 2025, 180 employees enrolled for a 30 min consultation including medical history review, lifestyle assessment, and evaluation of vaccination and screening status according to age, risk factors, and national guidelines. Standardized tools (PSS-14, PHQ-9) assessed perceived stress and mental well-being. Participants rated satisfaction and perceived care quality on a 10-point Likert scale. **Results**: Of 180 enrolled, 154 completed the evaluation. The majority of participants were female (68.8%), with a mean age of 54 years, and 42.9% held a higher education degree. Common lifestyle characteristics included current smoking (24.7%), regular alcohol consumption (9.8%), and insufficient sleep (mean 6.5 h/night). Overweight (40.3%) and obesity (29.2%) were prevalent. Chronic conditions were reported in 87.0% of participants, with dyslipidemia (54.5%), allergies (35.8%), and hypertension (26.9%) being the most frequent. Criteria for metabolic syndrome were found in 33.1% of participants with a higher prevalence in men (50.0% vs. 25.0%; *p* = 0.029). Mental health assessments revealed moderate stress levels (mean PSS-14: 23.7) and mostly minimal depressive symptoms (mean PHQ-9: 4.3). Preventive screening was variable, with higher adherence for mammography (79.2%) and lower for colonoscopy (40.2%). Service satisfaction was high, with significant increases in perceived usefulness (8.96 to 9.80, *p* < 0.001) and satisfaction (9.08 to 9.87, *p* < 0.001) after the intervention. **Conclusions**: This pilot revealed critical gaps in vaccination, cardiometabolic risk, and stress management among public employees. It was also shown that integrated workplace-based health models are both feasible and acceptable. These models can effectively deliver preventive actions on a scale and represent a promising strategy for strengthening occupational health in employed adult population.

## 1. Introduction

In recent decades, the health of working-age populations has become a growing priority in public health agendas worldwide, driven by the increasing burden of chronic diseases, mental health conditions, and modifiable risk behaviors [1]. The workplace—where adults spend a significant portion of their lives—has emerged as a key setting for implementing preventive services, promoting health literacy, and facilitating early detection of health risks. The World Health Organization (WHO) recognizes the workplace as a vital arena for health promotion, recommending targeted interventions that support disease prevention, stress management, and healthy lifestyle adoption [2]. Effective workplace health programs have been linked to improved quality of life, reduced absenteeism, increased productivity, and decreased long-term healthcare costs [3,4].

Despite this growing body of evidence, many countries still lack integrated and systematic approaches to workplace health—especially within the public sector. Occupational health services often remain limited to regulatory compliance and administrative procedures, neglecting the broader spectrum of preventive care and employee well-being [5]. This gap is particularly evident in Greece, where healthcare system reforms in the past decade, have largely focused on hospital-based care, leaving primary care and preventive services not fully implemented [6].

In the Greek public sector, occupational health is typically reactive rather than preventive or proactive. Services are fragmented, and the division between primary care and occupational medicine results in missed opportunities for timely interventions such as vaccinations, routine screenings, and behavioral counseling [7]. Public employees frequently face challenges in accessing preventive care during working hours, leading to underutilization of essential services and unaddressed health risks. Chronic stress, sedentary lifestyles, smoking, and poor dietary habits are common among this population, contributing to increased risk for non-communicable diseases (NCDs) such as cardiovascular disease, type 2 diabetes, and cancer [8]. Moreover, adult immunization rates remain below national and international targets, and mental healthcare is rarely integrated into workplace settings [9]

The COVID-19 pandemic further underscored these vulnerabilities. It highlighted the urgent need for more accessible workplace-based services, especially those related to adult immunization, psychosocial support, and chronic disease prevention [10,11,12,13]. This was also observed among Greek citizens, where despite moderate awareness in areas such as transmission routes, notable gaps persist in demographic subgroups indicating that health-related knowledge is not consistently high [14]. While Greece’s National Public Health Plan 2020–2025 sets targets for vaccinations and screenings, these priorities have yet to be systematically embedded in workplace contexts [15,16]. Public employees—a stable and accessible population—represent a critical group in which integrated, preventive health models can be piloted and evaluated.

Globally, integrated care models that combine occupational health with primary and public health strategies are gaining attention. These models offer streamlined risk assessments, evidence-based screenings, lifestyle interventions, and follow-up mechanisms, and have been shown to improve key indicators such as smoking cessation, physical activity, and chronic disease control [17,18]. Workplace-based mental health interventions using standardized tools like the Perceived Stress Scale (PSS-14) and the Patient Health Questionnaire (PHQ-9) are also effective in reducing psychological distress and enhancing employee resilience [19]. However, in Greece, such models remain rare and under-researched, with little localized evidence to inform their design or implementation [20,21]. For example, although Greece’s National Public Health Plan 2020–2025 includes preventive screening actions these are not embedded in workplace health systems and depend heavily on individuals accessing external services. Hence, coverage rates for vaccines remain below recommended thresholds [22,23,24]. Similarly, preventive screenings for metabolic and oncological risk factors or other chronic diseases (i.e., respiratory disorders) are provided based mostly on academic initiatives [25,26,27,28,29,30]. Furthermore, mental health, despite its importance as well as its intricate link with physical illnesses is poorly integrated into occupational health practice and public employees frequently report elevated stress levels, yet systematic assessment and intervention strategies are absent from the workplace [31,32,33].

This pilot study introduces and evaluates an integrated “mixed service” model in the Region of Crete. The model combines elements of occupational health and primary care into a unified, workplace-based intervention for public employees. Τhe initiative includes short clinical consultations (~30 min), lifestyle risk assessments, immunization reviews, mental health screening, and tailored follow-up recommendations. By leveraging the administrative structure and access mechanisms of the Region, the model seeks to deliver preventive services in a convenient and responsive format.

This prospective observational cohort study aimed to identify individual gaps in preventive healthcare among public sector employees and implement tailored interventions in response. The objectives of this study are to describe the design and operational characteristics of the pilot model, to present findings regarding the health status, risk factors, and unmet needs of public employees; and to assess the feasibility and relevance of such a model for wider adoption in the Greek health system. By integrating biomedical, and psychosocial information in a real-world workplace context, this intervention aims to contribute new evidence toward the development of effective, sustainable workplace health strategies in Greece and other resource-constrained health services.

## 2. Materials and Methods

### 2.1. Study Design

The study was conducted by a multidisciplinary team affiliated with the Department of Social Medicine, School of Medicine, University of Crete. Participants were followed through a structured protocol involving questionnaire-based assessment, biometric and clinical data collection, and follow-up sessions where applicable.

### 2.2. Ethical Approval and Informed Consent

The study protocol was reviewed and approved by the Ethics Committee of UoC (Approval Number: 131; Date: 4 November 2024). All participants were informed of the study’s objectives, procedures, and voluntary nature and provided written informed consent prior to participation. The study complies with the Declaration of Helsinki and relevant national and institutional research ethics guidelines.

### 2.3. Setting and Participants

Participants were appointed civil servants employed by the Regional Unit of Heraklion, Region of Crete, Greece. Participants eligible for the study were adults aged 18 years or older, employed on a permanent basis by the Regional Unit of Heraklion included in the current phase. Individuals who declined participation or could not be reached after multiple contact attempts were not included in the study sample.

A total of 366 potential participants were actively appointed to be enrolled in the study between January and July 2025. Recruitment was initiated through an informational email circulated by the Employee Association of the Regional Unit of Heraklion. Subsequently, participants were contacted individually via their workplace telephone numbers, provided by the same administrative body. During these calls, the study’s aims and procedures were explained, and appointments were scheduled. Participants were asked to bring results from any clinical tests conducted within the past year to facilitate the development of a comprehensive baseline health profile.

### 2.4. Data Collection Procedures

Data collection was conducted prospectively between January and July 2025 through scheduled 30 min appointments using the described standardized procedures to ensure consistency. All interviews were conducted in a designated space provided and equipped by the Region of Crete. Upon arrival, participants’ identities were confirmed, and healthcare professionals introduced themselves and described their roles in the study. At the outset, participants were asked to complete a brief four-domain service evaluation form. The first two items assessed expectations and were completed before the consultation, while the remaining two items, focusing on the experience, were completed afterward.

A member of the healthcare team initiated the consultation and began completing the sections of the questionnaire through a structured dialogue with the participant. Biometric data, including blood pressure (BP), heartbeats per minute (HPM), oxygen saturation, height, weight, and body mass index (BMI), were measured on-site using standardized equipment. If participants presented recent laboratory test results regarding complete blood count (CBC) and comprehensive metabolic panel (CMP), these were recorded. In cases where test results were outdated or incomplete for constructing a comprehensive health profile, the participant was advised to undergo additional testing and was issued the necessary referrals. Laboratory test results older than 4 months at the time of the initial interview were deemed outdated, and new or supplementary tests were prescribed accordingly. Dyslipidemia was defined according to the NCEP ATP III criteria: total cholesterol ≥ 200 mg/dL, LDL-C ≥ 130 mg/dL, HDL-C < 40 mg/dL in men or <50 mg/dL in women, or triglycerides ≥ 150 mg/dL, or current use of lipid-lowering medication [34]. These criteria were applied to cases not previously diagnosed with dyslipidemia and in which participants were not aware of their condition. Hypertension was defined by one or more of the following: (i) systolic blood pressure ≥ 140 mmHg or diastolic blood pressure ≥ 90 mmHg measured in the office setting according to ESH guidelines; (ii) self-reported use of antihypertensive medication. Blood pressure measurements were performed following the 2021 ESH practice guidelines for office measurement to ensure accuracy. Allergy was defined as a participant’s self-reported history of a physician-addressed allergic condition, including drug, food, or environmental allergies.

Immunization status was initially self-reported by participants in response to structured questions and then cross-verified through their health records according to the national adult immunization program [35]. Mental health was assessed using the validated Greek versions of the PSS-14 (Cronbach’s α = 0.85) and the PHQ-9 (Cronbach’s α = 0.75) [19,36,37,38]. Both instruments were completed independently by participants after a brief explanation. In cases of elevated stress or depressive symptoms, interventions were proposed immediately, ranging from education and counseling to support or referral to specialist mental health services. Metabolic syndrome (MetS) risk was evaluated using the National Cholesterol Education Program’s Adult Treatment Panel III (NCEP ATP III, 2005 revision) criteria, including BP, fasting glucose, triglycerides, HDL cholesterol, and waist circumference.

The consultation concluded with the formulation of a personalized action plan. Any intervention such as screenings, referrals, prescriptions, or counseling—were documented in the attendee’s record. A signed record of agreed-upon actions was obtained from both the participant and the attending health professional. Follow-up visits were scheduled as needed based on nature and the number of interventions.

### 2.5. Intervention and Follow-Up Procedures

The interventions implemented in this pilot were personalized and based on the specific needs identified during the initial health assessment. These included the prescription of diagnostic examinations such as mammography, low-dose chest CT, or comprehensive blood panels. When gaps in vaccination coverage were identified, appointments were arranged to facilitate immunization in accordance with national adult vaccine recommendations. Participants also received tailored counseling on key health behaviors, including dietary modification, physical activity enhancement, and smoking cessation. When clinical indicators suggested the need for further evaluation, participants were referred to appropriate specialists such as cardiologists, endocrinologists, or mental health providers. Additionally, educational materials and individualized behavior change plans were provided to support long-term health improvement.

Follow-up sessions were arranged according to the type and number of interventions planned for each participant. These sessions typically lasted 15 to 30 min and served multiple purposes, including reviewing diagnostic results, verifying completed vaccinations, reassessing behavioral changes, and modifying or extending care plans when necessary. Once all identified preventive care gaps had been adequately addressed and the planned interventions were completed, the participant’s file was marked as complete, and no further visits were scheduled unless novel issues arose.

### 2.6. Service Satisfaction Assessment

Perceived service satisfaction was evaluated using a brief, structured tool consisting of two 10-point Likert-scale items. The first item, administered before the consultation, assessed participants’ expectations regarding the quality and usefulness of the health service. The second item, administered immediately after the consultation, captured participants perceived level of satisfaction with the care received. Both items used a scale from 1 (very low) to 10 (very high). This pre–post format allowed for a basic comparison between initial expectations and actual experience, providing insight into the acceptability and perceived value of the intervention model from the user’s perspective.

### 2.7. Statistical Analysis

Data analysis was conducted using the SPSS program (IBM Corp. Released 2019, IBM SPSS Statistics for Windows, v.25.0, Armonk, NY, USA: IBM Corp.). Frequency distributions and measures of location and dispersion of the characteristics of the 154 participants were calculated. The prevalence of chronic conditions, vaccination status and proactive action/recommendation/prescription was also estimated as well as the levels and frequencies of the five MetSyn risk factors. Gender differences were detected based on Student *t* and χ^2^ tests. Score levels of PSS-14, PHQ-9 and satisfaction scales regarding the Pro-vision and Reception of Services from the Current Health Assessment were assessed for normality using Blom’s (Q-Q plot) method. In satisfaction scale, the paired sample Student t test was used to detect any significant change between expected and perceived status. Multiple logistic regression analysis was also implemented of the in-creased levels of PSS-14 and PHQ-9 scales of study’s participants, and in relation to their basic characteristics, health habits and the presence of metabolic syndrome (MetS). The acceptable level of significance was set at 0.05.

## 3. Results

### 3.1. Participation and Demographics

Of the 366 employees eligible for participation, 74 were not reached despite multiple contact attempts thus leaving 292 eligible participants. Of them, till 10 July, 180 (67.7%) agreed to schedule an evaluation appointment, and 154 ultimately attended, yielding a response rate of 85.6%. Among those who did not participate, 58 individuals declined the invitation and 54 expressed willingness to arrange a later appointment. Table 1 summarizes the demographic characteristics of the 154 participants and their health habits. The majority of participants were female (68.8%), with a mean age of 54 years (±6.1), and 42.9% held a higher education degree. In terms of health habits, 24.7% of participants were current smokers, reporting a mean smoking of 14 cigarettes per day over a mean smoking duration of 27 years. Approximately 10% of participants reported regular alcohol consumption. The mean reported sleep duration was 6.5 h per night. The mean BMI was 28.3 (±5.8), with 40.3% classified as overweight and 29.2% as obese.

### 3.2. Prevalence of Chronic Conditions and Metabolic Syndrome

**Figure 1:**
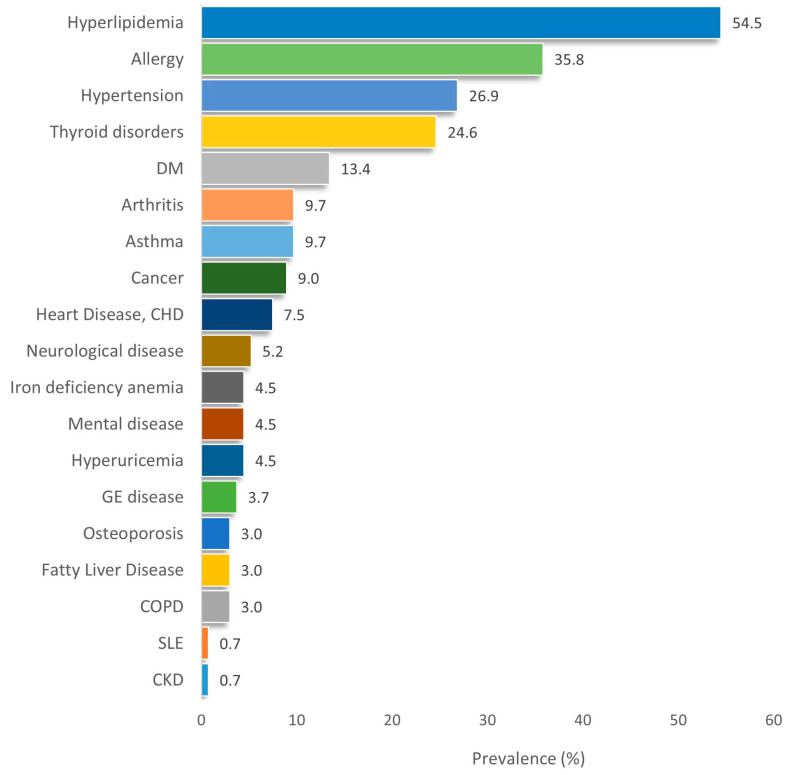
Prevalence of chronic conditions among the study’s participants. (Abbreviations: DM: Diabetes Melitus; CHD: Coronary Heart Disease; GE: gastroenterological disease; COPD: Chronic Obstructive Pulmonary Disease; SLE: Systemic Lupus Erythematosus; CKD: Chronic Kidney Disease).

Among the 154 participants assessed, chronic conditions were common in 134 of them. Figure 1 illustrates their frequencies as were recorded in the current study. Among 19 chronic conditions, dyslipidemia was with the higher prevalence (54.5%) followed by allergies (35.8%) or hypertension (26.9%).

A focused subgroup analysis was conducted on 130 participants with complete laboratory and biometric data to evaluate MetS risk factors based on the NCEP ATP III criteria (Table 2). Mean values for MetS-related parameters were generally close to the defined diagnostic thresholds. A substantial proportion of participants presented with abnormal values, including elevated BP (72.3%), increased fasting glucose (35.4%), and excessive waist circumference (59.2%). Although mean levels of BP, HDL-C, and waist circumference differed between males and females, only the proportion of individuals with increased waist circumference was significantly higher among males (*p* = 0.019). Overall, 33.1% of the subgroup met the criteria for metabolic syndrome, with prevalence significantly higher in males compared to females (50.0% vs. 25.0%; *p* = 0.029).

### 3.3. Vaccination Status

Vaccination coverage was suboptimal across key adult immunizations (Table 3). Although 61.0% of participants reported completion of the primary tetanus–diphtheria–pertussis (Td/Tdap/Tdap-IPV) series, only 27.9% had received a booster between the ages of 18 and 25, and just 11.0% had received a booster within the past decade. Pneumococcal vaccination, with PCV20 or earlier formulations such as PCV13 and PPSV23, had been administered in only 17.2% of participants. This estimate was based on the eligibility criteria of the national adult immunization program, which recommends vaccination for all adults aged ≥65 years and for those aged 18–64 years with chronic medical conditions such as chronic heart, lung, or liver disease, diabetes mellitus, alcoholism, asplenia, cerebrospinal fluid leaks, presence of cochlear implants or immunocompromising states. Furthermore, from the suitable for pneumococcal vaccine, 14 individuals declined discussion reflecting an element of vaccine hesitancy within the group. Coverage with the recombinant zoster vaccine (RZV), recommended for adults over 60 years of age or for immunocompromised individuals, was similarly low, with only 23.1% of eligible participants having received it. Again, from those that had not received it, two individuals declined discussion as to the necessity of the vaccination.

### 3.4. Mental Health and Perceived Stress

Data from 154 participants were analyzed using the PSS-14 and PHQ-9 tools (Table 4). The mean PSS-14 score was 23.7, indicating moderate stress levels within the participants. Based on standard cutoffs, 6.6% of participants seem to have low stress, 58.3% moderate, and 35.1% high stress levels. The PHQ-9 yielded a mean score of 4.3 or minimal mean levels. Approximately 60.9% reported minimal or no depressive symptoms, 28.5% mild symptoms, and 10.6% moderate to severe levels of depressive symptomatology.

### 3.5. Diagnostic and Preventive Action Patterns

**Figure 2:**
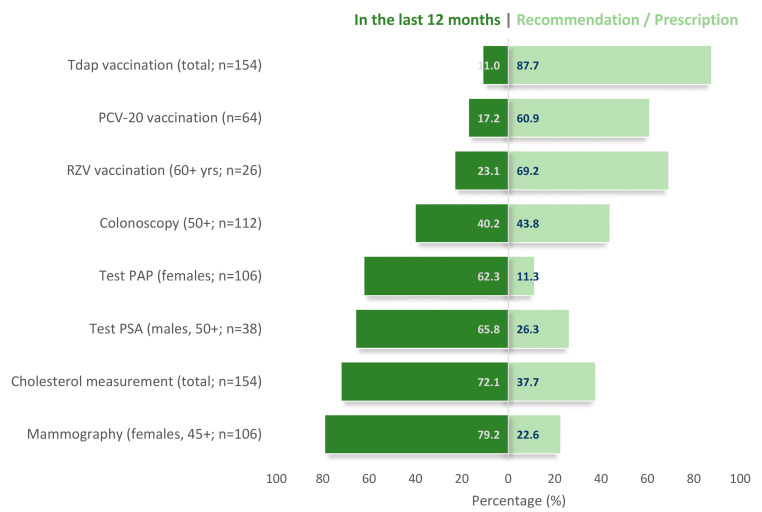
Frequency and trends of diagnostic tests or check-ups in the last 12 months (green) over recommendations/prescriptions for proactive action during interview (light green) among study’s participants. Sufficient trends exist between certain proactive actions. Percentages are calculated according to the number of individuals in each subgroup. Deviations from 100% are due to either pending appointments with their own doctor, testing on individual demand, or denial to adopt specific advice. (Tdap: Tetanus, Diphtheria, and Acellular Pertussis vaccine; PCV: Pneumococcal Conjugate Vaccine; RZV: Recombinant Zoster Vaccine; Pap test: Papanicolaou test (commonly called Pap smear); PSA test: Prostate-Specific Antigen test).

Figure 2 illustrates the balance between previously addressed (over the past 12 months) and additional preventive needs across key domains, including adult vaccinations (e.g., Tdap, PCV20, RZV), cancer screenings (e.g., colonoscopy, Pap test, prostate-specific antigen PSA, mammography), and cardiometabolic assessments (cholesterol measurement). All interventions were individualized according to subgroup-specific requirements, including age, gender, clinical history, risk factor profiles, and personal concerns. The figure illustrates characteristic trends regarding the tendency of compliance with specific preventive examinations versus preventive interventions such as vaccinations in which the frequency for recommendations was higher.

Uptake varied across interventions. Mammography and Pap smears demonstrated the highest completion rates (79.2% and 62.3%, respectively), while 22.6% and 11.3% of women, respectively, still required updated screening. PSA testing among men aged ≥50 years showed a completion rate of 65.8%, with 26.3% receiving a recommendation. Colonoscopy among eligible participants (≥50 years) had lower uptake, with only 40.2% having undergone the procedure and 43.8% newly advised to do so. For metabolic assessments, cholesterol measurement had been performed in 72.1% of participants, with 37.7% receiving new prescriptions, reflecting increased monitoring and need for recheck for some people. In terms of immunizations, coverage was notably low. Only 11.0% of participants had received a Tdap booster in the last 10 years, with 87.7% newly recommended. Similarly, 17.2% had received pneumococcal vaccination (PCV20 or earlier), with 60.9% newly recommended. Among those eligible for recombinant zoster vaccination (RZV; ≥60 years or immunocompromised), just 23.1% had received it, and 69.2% were advised to do so.

Overall, the workplace consultation prompted a substantial number of preventive health actions, as provided in Table 5. A total of 46 distinct interventions were documented, yielding 1079 actions across 154 participants, with a mean and median of 7.0 actions per person (range: 0–18) (see Supplementary Table S2 for details). Vaccination was the most frequent preventive measure, recommended to 91.6% of participants. Biochemical blood tests followed (68.8%), most often including HbA1c, kidney and liver function panels, fasting glucose, lipid profiles, and complete blood counts. Lifestyle counseling was also widely emphasized (63.0%), covering areas such as weight management, stress reduction, and smoking cessation. Socio-demographic screenings and preventive tests were suggested for 44.8% of participants, with cancer prevention measures—such as colonoscopy, mammography, PSA testing, and Pap smears—standing out. Pharmacological prescriptions were provided to 31.8%, while other targeted actions (9.1%) included spirometry, bone density scans, vitamin D supplementation, mental health referrals, low-dose CT scans, osteoporosis screening, and specialist evaluations.

### 3.6. Service Satisfaction

**Figure 3:**
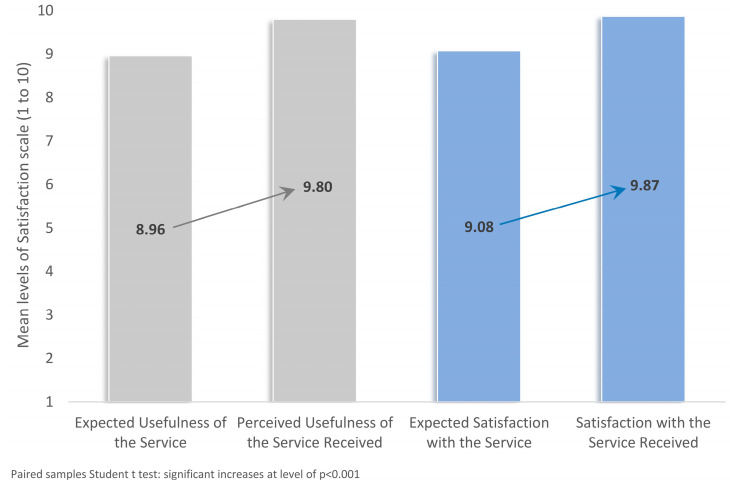
Mean levels of satisfaction scale regarding the Provision and Reception of Services from the Current Health Assessment of study participants.

Healthcare service satisfaction was evaluated using two 10-point Likert-scale questions administered before and after the clinical consultation (Figure 3). The mean score for expected usefulness of the service prior to the visit was 8.96, while the perceived usefulness after the session increased significantly to 9.80 (*p* < 0.001). Likewise, the average rating for expected satisfaction before the consultation was 9.08, which rose significantly also to 9.87 following the assessment (*p* < 0.001).

## 4. Discussion

### 4.1. Main Findings in the Context of Existing Literature

This study evaluated the feasibility and preliminary outcomes of a novel mixed health service model that integrates occupational health with primary preventive care among public sector employees in Crete, Greece. To the best of our knowledge, this is one of the first initiatives in Greece to combine occupational health data with primary preventive care actions. The study highlights critical gaps in preventive health behaviors, cardiometabolic risk, adult immunization and in lesser extent in mental well-being. Furthermore, the study reveals a strong degree of acceptability, engagement, and satisfaction with the intervention—both immediately and quantitatively—suggesting the model’s viability in workplace settings.

The distribution of preventive actions revealed clear differences in perceived importance and acceptance among employees of proactive health actions (Figure 2). Certain measures showed high completion rates (i.e., mammography) and thus appear to be well-established and “popular” preventive practices within this group. Others (i.e., colonoscopy) were more evenly distributed between prior completion and new recommendations, suggesting moderate uptake but also considerable room for improvement. By contrast, adult vaccinations (Tdap, PCV20, RZV), although simple actions with no expected outcome uncertainty displayed the lowest prior coverage revealing a negligent attitude despite their clear preventive value. These patterns highlight the heterogeneous attitudes toward prevention, ranging from well-accepted to poorly accepted actions. Another key observation was the high prevalence of modifiable behavioral and metabolic risk factors. Smoking remains prevalent (24.7%), and over 70% of participants were overweight or obese. These findings are consistent with national and European data indicating elevated rates of cardiovascular risk factors among adults [39,40,41,42,43]. More importantly, 36.8% of participants met three or more criteria for metabolic syndrome, highlighting a latent burden of cardiometabolic risk factors. This finding reinforces the notion of a ‘silent epidemic’ of cardiometabolic and other non-communicable diseases—particularly in urban settings where sedentary lifestyles and chronic stress are prevalent—mirroring trends observed in Mediterranean and rapidly urbanizing populations [22].

Vaccination coverage was also suboptimal. TdaP coverage was insufficient to meet recommended levels. Pneumococcal and herpes zoster vaccination rates were particularly low, reflecting national patterns of underutilization of adult immunization services in Greece [16,22]. Notably, 14 participants for PCV20 and 2 for RZV expressed hesitancy or outright refusal to consider vaccines reflecting the often-observed distrust in vaccination programs and highlight vaccine hesitancy as a critical barrier to achieving optimal coverage [44,45]. The gap between immunization guidelines and actual uptake underscores missed opportunities that could be addressed more effectively through workplace-based programs that promote health literacy and address factors contributing to vaccine hesitancy [46]. This issue is especially relevant in the post-COVID-19 context, where workplace-based health services may serve as important yet underutilized platforms for promoting adult vaccination [47,48,49].

Mental health emerged also as a major concern in the current analysis. Based on PSS-14 results, one-third of participants reported high levels of perceived stress, while PHQ-9 scores revealed mild to moderate depressive symptoms in a notable subset of the population. These rates are comparable to those reported in European public workforce studies, where job-related stress and burnout remain prevalent, especially in civil service sectors [50,51,52]. Global evidence supports the inclusion of mental health components in workplace interventions, with proven benefits for employee well-being and productivity [52]. Our results support growing evidence that mental health screening tools like the PSS-14 and PHQ-9 are feasible and effective in identifying distress in occupational settings. However, such tools are rarely deployed in standard occupational health programs in Greece. Integrating routine mental health assessments into workplace models could help fill this gap while also reducing stigma and facilitating timely referrals.

In terms of intervention delivery, the mixed health service model proved both feasible and efficient. On average, 7 interventions were delivered per attendee, spanning 46 distinct types of interventions such as immunizations, lifestyle counseling, and chronic disease risk screening. This volume of interventions indicates a substantial latent demand for preventive services among public employees and reinforces the argument that workplace-based screening can function as a gateway to the broader health system. The comprehensiveness of care offered aligns well with best practices worldwide, which advocate for multicomponent, individualized workplace health promotion programs [53]. A particularly notable finding was the high level of satisfaction reported by participants following the health consultations. The mean post-consultation satisfaction scores were significantly higher than pre-consultation expectations (*p* < 0.001), highlighting both the acceptability of the model and the value placed on personalized, accessible care. This result aligns with prior studies showing that when workplace health services are well-structured, respectful, and informative, they tend to generate strong user engagement and satisfaction—critical factors for program sustainability and policy adoption [54,55,56,57,58].

Overall, this model addresses notable gaps in Greece’s occupational health system, offering a pragmatic strategy for integrating primary prevention into routine workplace health services. It supports WHO recommendations for embedding preventive care in occupational settings and could serve as a scalable framework for similar public sector contexts, both nationally and internationally [59,60,61].

### 4.2. Strengths, Limitations, and Directions for Future Research

This study introduces an innovative approach to preventive health by embedding primary care competencies into the occupational health framework [62,63,64]. High initial willingness to participate (180 out of 366 employees) indicates feasibility and acceptability, especially given the traditionally low engagement with preventive health services among working populations. Key methodological strengths include the use of validated screening tools (e.g., PSS-14, PHQ-9), multidimensional health profiling (behavioral, metabolic, immunization, and psychosocial parameters), and the provision of 46 distinct interventions highlighting the model’s adaptability to individual health needs [65,66,67].

However, some limitations must be acknowledged. As a single-site pilot study, the preliminary and localized nature of this analysis limits generalizability. The absence of a control group restricts causal inference, while the cross-sectional design precludes conclusions about long-term effectiveness. Additionally, variables, such as smoking status, relied on self-report and are subject to recall bias. Objective clinical outcomes beyond BMI and metabolic markers were not captured, representing another limitation. Operational challenges, including scheduling constraints and limited follow-up capacity, may also affect participation rates and sustainability, particularly in resource-limited public sector environments.

Future research should focus on longitudinal follow-up to assess the sustained impact of the mixed health service model on employee health outcomes, healthcare utilization, and economic indicators such as absenteeism and productivity. While this pilot study offers encouraging initial results, it remains essential to determine whether these improvements are maintained over time and whether they translate into reduced long-term healthcare costs. Evaluating the model’s scalability and cost-effectiveness in diverse public sector settings across Greece would provide critical insights for broader implementation and inform national policy development. Upcoming studies should involve larger, more diverse groups across multiple regions to better capture variations in health behaviors and service needs. Longitudinal designs that track clinical and laboratory parameters such as blood pressure, lipid levels, HbA1c, and inflammatory markers would offer objective evidence of physiological benefits and improve risk stratification. Additionally, economic evaluations assessing cost-effectiveness and return on investment will be key to supporting long-term funding and policy integration of workplace health programs. A clear understanding of financial outcomes will help secure sustained support from public and private sector stakeholders [68].

The integration of digital health tools should also be explored. Till today there are examples of digital tools that can promote healthy lifestyle behaviors within communities [69,70,71,72,73]. Future research could investigate the use of mobile health apps or telehealth solutions to monitor health indicators in real time, facilitate ongoing employee engagement, and provide accessible platforms for delivering health interventions and support from healthcare providers [74,75,76]. Digital health solutions have the potential to increase the reach and sustainability of workplace health services [77].

Finally, policy implementation research should be conducted to investigate how workplace health programs can be effectively embedded within national and organizational health policy frameworks [78]. Understanding the barriers and facilitators to the integration of such programs will be crucial for scaling them up across different regions and sectors. Policymakers must ensure that these interventions are not only effective but also sustainable in the long term.

### 4.3. Implications for Practice and Policy

This study highlights the value of integrating preventive care into occupational health frameworks, particularly in public sector settings and the successful implementation of the mixed health service model carries important implications for both clinical practice and public health policy. The model proved both feasible and acceptable to employees, demonstrating its potential as a scalable solution for managing health risks and enhancing well-being. Embedding services such as health screenings, immunizations, and mental health evaluations within the workplace offers a more accessible and less stigmatizing approach to preventive care. This can lead to more comprehensive care, improved health outcomes, and reduced long-term healthcare costs. Occupational health services should extend beyond regulatory compliance and include routine screening, vaccination, and psychosocial support. The high satisfaction reported in this study suggests employees value personalized, accessible care. Tools such as the PSS-14 and PHQ-9 facilitate early identification of risk and enable tailored interventions for vulnerable individuals [79,80,81,82]. Mental health services and workplace-based interventions must become integral to occupational health, with dedicated referral pathways and anti-stigma initiatives to encourage help-seeking [83,84,85].

At the policy level, the findings underscore the need for legislative and organizational reforms that integrate workplace health services into the public sector while addressing barriers and implementation challenges [86]. Workplace health strategies must also be culturally adapted to support diversity, inclusivity, and equity [87]. Prioritizing employee well-being alongside performance goals creates mutual benefits for both staff and organizations [88]. Developing national or global workplace health standards through participatory processes could reduce inconsistencies and strengthen framework implementation across diverse settings [89]. In Europe, the WHO Health 2020 framework emphasizes addressing social determinants of health to reduce inequities, including those linked to workplace conditions [90]. U initiatives have advanced regulation and programs for promoting workplace well-being, but uptake remains inconsistent, and implementation limited. Clear standards for regular screenings, vaccination campaigns, and psychosocial support within occupational health regulations are therefore essential [91,92,93]. Focus should be placed on interventions with low uptake but high potential impact, such as adult vaccination and cancer screening, while maintaining widely accepted practices like mammography and biochemical monitoring [94,95,96]. Attention should also be given to links between social determinants and physical phenotypic expressions [97]. Tailored communication, improved health literacy, and systematic follow-up mechanisms are key to closing preventive care gaps. Finally, policies that incentivize or mandate workplace health promotion could embed these practices into organizational operations, ensuring consistent access across regions and departments [98,99,100,101].

Sustained public investment in workplace health programs is essential. Dedicated funding should support both implementation and training for occupational health professionals, ensuring delivery of comprehensive, high-quality care [102,103,104]. Resources should also support the integration of digital health tools that can enhance monitoring and employee engagement [105,106]. Digital platforms offer added value by fostering collaboration between healthcare providers, employers, and insurers, and by empowering citizens at the community level to access evidence-based care [75,107,108]. Policies encouraging public–private partnerships could improve coordination, resource sharing, and data integration, ultimately reduce fragmentation, and enhance continuity of care [109,110]. Moreover, policies must directly address key challenges identified in this study, such as low adult vaccination rates and high levels of work-related stress. Targeted public health campaigns and mental health support services should be expanded within workplaces to improve immunization uptake and psychosocial well-being [111]. Given the high prevalence of stress and depressive symptoms, mental health should be prioritized within occupational health programs. This includes mandatory risk assessments, workplace support infrastructure, and anti-stigma efforts [112].

In conclusion, this study reinforces the potential of integrated, workplace-based preventive health models to improve employee well-being and reduce healthcare costs. Embedding such approaches into national strategies can strengthen workforce health, advance population health goals, and ensure sustainable improvements in public sector health outcomes.

## 5. Conclusions

The present study illustrates the feasibility, acceptability, and potential utility of a mixed health service model that bridges primary preventive care and occupational health within the public sector workforce. The integration of these services enabled early identification of unmet health needs—particularly those related to lifestyle risk factors, metabolic syndrome, inadequate immunization coverage, and high psychosocial stress—reflecting broader systemic gaps in adult preventive healthcare for Greece. By delivering targeted, individualized interventions during structured consultations, the model achieved a high intervention-to-participant ratio and elicited positive user feedback. These findings suggest that embedding preventive strategies into occupational settings is both a practical and impactful approach to improving employee health outcomes, particularly in systems where access to routine preventive care remains fragmented or underutilized. Nevertheless, the study is exploratory in nature, with limitations including a relatively small and localized sample and lack of a control group.”. Future research should focus on scaling this approach across broader geographic and institutional settings, incorporating longitudinal impact assessments, cost-effectiveness evaluations, and exploring digital health tools to enhance service continuity and integration.

## Figures and Tables

**Table 1:** Demographic characteristics of 154 participants in the current study.

			n	%
Gender	males/females		48/106	31.2/68.8
Age, years	mean ± stand. dev.		54.0 ± 6.1	
	50+ years		112	72.7
	60+ years		26	16.2
Education level	High school		25	16.2
	University/Technical School		66	42.9
	MSc		53	34.4
	PhD		10	6.5
Smoking	nonsmokers		80	51.9
	ex		36	23.4
	current		38	24.7
Cigarettes/day	median (IQR)		15 (10, 20)	
Years of smoking	median (IQR)		29 (20.0, 35.0)	
Alcohol consumption (at least half of drink per day)		yes	15	9.8
Sleep hours at night	mean ± stand. dev.		6.5 ± 1.2	
Body Mass Index, kg/m^2^	mean ± stand. dev.		28.3 ± 5.8	
	Overweight/obese		62/45	40.3/29.2

MSc: A Master of Science; PhD: Doctor of Philosophy; IQR: Interquartile Range; stand. dev.: Standard deviation.

**Table 2:** Levels and frequencies of five MetS risk factors among 154 participants.

		Total (n = 130)	Males (n = 32)	Females (n = 88)	
Risk Factors & MetS Criteria ^a^		Mean ± Stand. dev.			*p*-Value ^b^
SBP (mmHg)		134.4 ± 17.4	143.0 ± 19.0	130.2 ± 15.0	<0.001
DBP (mmHg)		86.7 ± 11.6	89.8 ± 11.7	85.2 ± 11.4	0.037
>130/85		72.3%	83.3%	67.0%	0.052
Fasting glucose (mg/dL)		99.3 ± 15.3	102.4 ± 17.5	97.8 ± 14.0	0.103
≥100		35.4%	45.2%	30.7%	0.105
Triglycerides (mg/dL)		98.5 ± 47.3	108.2 ± 55.9	93.9 ± 42.2	0.106
≥150		13.1%	16.7%	11.4%	0.402
HDL cholesterol (mg/dL)		59.1 ± 12.8	52.5 ± 12.6	62.3 ± 11.6	<0.001
<40 (M), <50 (F)		16.9%	19.0%	15.9%	0.655
Waist circumference (cm)		98.3 ± 18.6	110.6 ± 17.9	92.4 ± 16.0	<0.001
>102 (M), >88 (F)		59.2%	73.8%	52.3%	0.019
MetS risk factors	none	15.4%	7.2%	19.3%	0.029
1		22.3%	19.0%	23.9%	
2		29.2%	23.8%	31.8%	
3+ or MetS		33.1%	50.0%	25.0%	

^a^ Based on the National Cholesterol Education Program’s Adult Treatment Panel III (NCEP ATP III—revision 2005) guidelines for MetS. ^b^ Student *t* and χ^2^ tests. Abbreviations: SBP systolic blood pressure; DSB: Diastolic Blood pressure.

**Table 3:** Vaccination status of study’s participants.

Vaccine Type		Vaccination Status	n	%
TD or Tdap or Tdap-IPV		Initial Vaccination	94	61.0
	Booster dose at age 18–25 with Tdap or Tdap-IPV		43	27.9
	Booster dose in the last decade with TD or Tdap		17	11.0
RZV (>60 years & immunocompromised)		no	20	76.9
		yes	6	23.1
PCV20 or prior (PCV13, PPSV23) *		no	53	82.8
		yes	11	17.2

* adults ≥ 65 years or 18–64 years with chronic conditions or immunocompromising states, as defined by the Greek National Vaccination Program. Abbreviations: Td/Tdap/Tdap-IPV: tetanus–diphtheria–pertussis vaccine; PCV20: 20-valent pneumococcal conjugate vaccine; RZV: recombinant zoster (shingles) vaccine.

**Table 4:** Score levels of PSS-14 and PHQ-9 scales of study’s participants.

Scales	Mean	Stand. Dev.	Median
PSS Score ^a^	23.7	7.4	24.0
low (0–13)	n = 10 or 6.6%		
moderate (14–26)	n = 88 or 58.3%		
high (27–42)	n = 53 or 35.1%		
PHQ-9 Score ^b^	4.3	4.3	3.0
none-minimal (0–4)	n = 92 or 60.9%		
mild (5–9)	n = 43 or 28.5%		
moderate to severe (10–27)	n = 16 or 10.6%		

^a^ Score was extracted summing up the responses of 14 items, ranging from 0 to 42. The higher the score the higher of perceived stress. ^b^ Score range 0–27. The higher the Score the worst of the severity of depression. Cutoffs proposed by Kroenke and colleagues (2001) [38].

**Table 5:** Preventive health actions as they suggested to study participants.

Proactive Action/Recommendation/Prescription	n	%
Vaccination	141	91.6
Biochemical blood test	106	68.8
Lifestyle Counseling	97	63.0
Socio-demographic screenings & preventive tests	69	44.8
Pharmacological intervention/prescription	49	31.8
Other actions	14	9.1

## Data Availability

The original contributions presented in this study are included in the article/Supplementary Material. Further inquiries can be directed to the corresponding authors.

## Supplementary Materials

The following supporting information can be downloaded at: https://www.mdpi.com/article/10.3390/healthcare13182337/s1, Table S1. Multiple logistic regression analysis of increased levels of Perceived Stress Scale (PSS-14) and Patient Health Questionnaire-9 (PHQ-9) scales of study’s participants, in relation to basic characteristics, health habits and the presence of metabolic syndrome (MetS). Table S2. Hierarchical frequency of 46 recorded intervention types (recommendations/prescription) in the current examination among the 154 participants.

## References

[1] (2014). Global Status Report on Noncommunicable Diseases. https://www.who.int/publications/i/item/9789241564854.

[2] Healthy Workplaces: A Model for Action. https://www.who.int/publications/i/item/9789241599313.

[3] Cancelliere C., Cassidy J.D., Ammendolia C., Côté P. (2011). Are Workplace Health Promotion Programs Effective at Improving Presenteeism in Workers? A Systematic Review and Best Evidence Synthesis of the Literature. BMC Public Health.

[4] Economou C., Kaitelidou D., Kentikelenis A., Maresso A., Sissouras A. (2015). The Impact of the Crisis on the Health System and Health in Greece. Economic Crisis, Health Systems and Health in Europe: Country Experience [Internet].

[5] Zavras D., Tsiantou V., Pavi E., Mylona K., Kyriopoulos J. (2013). Impact of Economic Crisis and Other Demographic and Socio-Economic Factors on Self-Rated Health in Greece. Eur. J. Public Health.

[6] Myloneros T., Sakellariou D. (2021). The Effectiveness of Primary Health Care Reforms in Greece towards Achieving Universal Health Coverage: A Scoping Review. BMC Health Serv. Res..

[7] Economou C. (2015). Barriers and Facilitating Factors in Access to Health Services in Greece.

[8] LaMontagne A.D., Keegel T., Louie A.M., Ostry A., Landsbergis P.A. (2007). A Systematic Review of the Job-Stress Intervention Evaluation Literature, 1990-2005. Int. J. Occup. Environ. Health.

[9] Eiden A.L., Barratt J., Nyaku M.K. (2022). Drivers of and Barriers to Routine Adult Vaccination: A Systematic Literature Review. Hum. Vaccin. Immunother..

[10] Guglielmi V., Colangeli L., Parrotta M.E., Ciammariconi A., Milani I., D’Adamo M., Sbraccia P., Capoccia D. (2025). Social Isolation and Loneliness in Non-Communicable Chronic Diseases: Impact of COVID-19 Pandemic, Population Aging and Technological Progress. Nutr. Metab. Cardiovasc. Dis..

[11] Filip R., Gheorghita Puscaselu R., Anchidin-Norocel L., Dimian M., Savage W.K. (2022). Global Challenges to Public Health Care Systems during the COVID-19 Pandemic: A Review of Pandemic Measures and Problems. J. Pers. Med..

[12] Overview of the Implementation of COVID-19 Vaccination Strategies and Deployment Plans in the EU/EEA. https://www.ecdc.europa.eu/en/publications-data/overview-implementation-covid-19-vaccination-strategies-and-deployment-plans.

[13] Vardavas C., Nikitara K., Aslanoglou K., Lagou I., Marou V., Phalkey R., Leonardi-Bee J., Fernandez E., Vivilaki V., Kamekis A. (2023). Social Determinants of Health and Vaccine Uptake During the First Wave of the COVID-19 Pandemic: A Systematic Review. Prev. Med. Rep..

[14] Dermitzakis I., Evangelidis N., Evangelidis P., Anestis A. (2021). Knowledge, Attitudes, and Perceptions Regarding COVID-19 Outbreak in Greece in September 2020: A Cross-Sectional Web-Based Survey. Hippokratia.

[15] Galanis P., Moisoglou I., Vraka I., Siskou O., Konstantakopoulou O., Katsiroumpa A., Kaitelidou D. (2021). Predictors of COVID-19 Vaccine Uptake in Healthcare Workers: A Cross-Sectional Study in Greece. J. Occup. Environ. Med..

[16] Bouloukaki I., Christoforaki A., Christodoulakis A., Krasanakis T., Lambraki E., Pateli R., Markakis M., Tsiligianni I. (2023). Vaccination Coverage and Associated Factors of COVID-19 Uptake in Adult Primary Health Care Users in Greece. Healthcare.

[17] Tan L., Wang M.J., Modini M., Joyce S., Mykletun A., Christensen H., Harvey S.B. (2014). Preventing the Development of Depression at Work: A Systematic Review and Meta-Analysis of Universal Interventions in the Workplace. BMC Med..

[18] Lassen A.D., Fagt S., Lennernäs M., Nyberg M., Haapalar I., Thorsen A.V., Møbjerg A.C.M., Beck A.M. (2018). The Impact of Worksite Interventions Promoting Healthier Food and/or Physical Activity Habits among Employees Working ‘around the Clock’ Hours: A Systematic Review. Food Nutr. Res..

[19] Cohen S., Kamarck T., Mermelstein R. (1983). A Global Measure of Perceived Stress. J. Health Soc. Behav..

[20] Antoniou M., Fradelos E.C., Roumeliotaki T., Malli F., Emmanouil K.S., Papagiannis D. (2024). Assessing Mental Resilience with Individual and Lifestyle Determinants among Nursing Students: An Observational Study from Greece. AIMS Public Health.

[21] Lionis C., Symvoulakis E.K., Markaki A., Petelos E., Papadakis S., Sifaki-Pistolla D., Papadakakis M., Souliotis K., Tziraki C. (2019). Integrated People-Centred Primary Health Care in Greece: Unravelling Ariadne’s Thread. Prim. Health Care Res. Dev..

[22] Tsiligianni I., Bouloukaki I., Papazisis G., Paganas A., Chatzimanolis E., Kalatharas M., Platakis I., Tirodimos I., Dardavesis T., Tsimtsiou Z. (2023). Vaccination Coverage and Predictors of Influenza, Pneumococcal, Herpes Zoster, Tetanus, Measles, and Hepatitis B Vaccine Uptake among Adults in Greece. Public Health.

[23] Tsachouridou O., Georgiou A., Naoum S., Vasdeki D., Papagianni M., Kotoreni G., Forozidou E., Tsoukra P., Gogou C., Chatzidimitriou D. (2019). Factors Associated with Poor Adherence to Vaccination against Hepatitis Viruses, Streptococcus Pneumoniae and Seasonal Influenza in HIV-Infected Adults. Hum. Vaccin Immunother..

[24] Papagiannis D., Rachiotis G., Mariolis A., Zafiriou E., Gourgoulianis K.I. (2020). Vaccination Coverage of the Elderly in Greece: A Cross-Sectional Nationwide Study. Can. J. Infect. Dis. Med. Microbiol..

[25] Koloverou E., Panagiotakos D.B., Pitsavos C., Chrysohoou C., Georgousopoulou E.N., Pitaraki E., Metaxa V., Stefanadis C. (2014). 10-Year Incidence of Diabetes and Associated Risk Factors in Greece: The ATTICA Study (2002–2012). Rev. Diabet. Stud..

[26] Kollia N., Panagiotakos D.B., Chrysohoou C., Georgousopoulou E., Tousoulis D., Stefanadis C., Papageorgiou C., Pitsavos C. (2018). Determinants of Healthy Ageing and Its Relation to 10-Year Cardiovascular Disease Incidence: The Attica Study. Cent. Eur. J. Public Health.

[27] Petridou E., Syrigou E., Toupadaki N., Zavitsanos X., Willett W., Trichopoulos D. (1996). Determinants of Age at Menarche as Early Life Predictors of Breast Cancer Risk. Int. J. Cancer.

[28] Faka A., Damigou E., Chrysohoou C., Barkas F., Vlachopoulou E., Dalmyras D., Vafia C., Michelis E., Loukina A., Mentzantonakis G. (2023). Twenty-Year Incidence Rates of Cardiovascular Disease in Greece: A Geospatial Analysis in the Attica Study Context, 2002–2022. J. Atheroscler. Prev. Treat..

[29] Kapantais E., Tzotzas T., Ioannidis I., Mortoglou A., Bakatselos S., Kaklamanou M., Lanaras L., Kaklamanos I. (2006). First National Epidemiological Survey on the Prevalence of Obesity and Abdominal Fat Distribution in Greek Adults. Ann. Nutr. Metab..

[30] Tzanakis N., Koulouris N., Dimakou K., Gourgoulianis K., Kosmas E., Chasapidou G., Konstantinidis A., Kyriakopoulos C., Kontakiotis T., Rapti A. (2021). Classification of COPD Patients and Compliance to Recommended Treatment in Greece According to GOLD 2017 Report: The RELICO Study. BMC Pulm. Med..

[31] Zagkas D.G., Chrousos G.P., Bacopoulou F., Kanaka-Gantenbein C., Vlachakis D., Tzelepi I., Darviri C. (2023). Stress and Well-Being of Greek Primary School Educators: A Cross-Sectional Study. Int. J. Environ. Res. Public Health.

[32] Baez A.S., Ortiz-Whittingham L.R., Tarfa H., Osei Baah F., Thompson K., Baumer Y., Powell-Wiley T.M. (2023). Social Determinants of Health, Health Disparities, and Adiposity. Prog. Cardiovasc. Dis..

[33] Kivimäki M., Nyberg S.T., Batty G.D., Fransson E.I., Heikkilä K., Alfredsson L., Bjorner J.B., Borritz M., Burr H., Casini A. (2012). Job Strain as a Risk Factor for Coronary Heart Disease: A Collaborative Meta-Analysis of Individual Participant Data. Lancet.

[34] National Cholesterol Education Program Expert Panel on Detection Evaluation and Treatment of High Blood Cholesterol in Adults (2002). Third Report of the National Cholesterol Education Program (NCEP) Expert Panel on Detection, Evaluation, and Treatment of High Blood Cholesterol in Adults (Adult Treatment Panel III) Final Report. Circulation.

[35] National Adult Vaccination Program 2025. Timetable and Recommendations. https://www.moh.gov.gr/articles/health/dieythynsh-dhmosias-ygieinhs/emboliasmoi/ethniko-programma-emboliasmwn-epe-enhlikwn/13219-ethniko-programma-emboliasmwn-enhlikwn-2025-xronodiagramma-kai-systaseis.

[36] Karekla M., Pilipenko N., Feldman J. (2012). Patient Health Questionnaire: Greek Language Validation and Subscale Factor Structure. Compr. Psychiatry.

[37] Katsarou A., Panagiotakos D., Zafeiropoulou A., Vryonis M., Papageorgiou C., Ioannis Skoularigis, Filippos Tryposkiadis (2012). Validation of a Greek Version of PSS-14; A Global Measure of Perceived Stress. Cent. Eur. J. Public Health.

[38] Kroenke K., Spitzer R.L., Williams J.B.W. (2001). The PHQ-9: Validity of a Brief Depression Severity Measure. J. Gen. Intern. Med..

[39] Iriti M., Varoni E.M., Vitalini S. (2020). Healthy Diets and Modifiable Risk Factors for Non-Communicable Diseases-The European Perspective. Foods.

[40] Valenzuela P.L., Santos-Lozano A., Saco-Ledo G., Castillo-García A., Lucia A. (2023). Obesity, Cardiovascular Risk, and Lifestyle: Cross-Sectional and Prospective Analyses in a Nationwide Spanish Cohort. Eur. J. Prev. Cardiol..

[41] Castillo-García A., Valenzuela P.L., Saco-Ledo G., Carrera-Bastos P., Ruilope L.M., Santos-Lozano A., Lucia A. (2024). Lifestyle and Cardiovascular Risk in Working Young Adults: Insights from a Nationwide Spanish Cohort. Rev. Española De Cardiol. íA (Engl. Ed.).

[42] Yusuf P.S., Hawken S., Ôunpuu S., Dans T., Avezum A., Lanas F., McQueen M., Budaj A., Pais P., Varigos J. (2004). Effect of Potentially Modifiable Risk Factors Associated with Myocardial Infarction in 52 Countries (the INTERHEART Study): Case-Control Study. Lancet.

[43] Gallucci G., Tartarone A., Lerose R., Lalinga A.V., Capobianco A.M. (2020). Cardiovascular Risk of Smoking and Benefits of Smoking Cessation. J. Thorac. Dis..

[44] Milionis C., Ilias I., Tselebis A., Pachi A. (2023). Psychological and Social Aspects of Vaccination Hesitancy—Implications for Travel Medicine in the Aftermath of the COVID-19 Crisis: A Narrative Review. Medicina.

[45] Goje O., Kapoor A. (2024). Meeting the Challenge of Vaccine Hesitancy. Cleve Clin. J. Med..

[46] Singh P., Dhalaria P., Kashyap S., Soni G.K., Nandi P., Ghosh S., Mohapatra M.K., Rastogi A., Prakash D. (2022). Strategies to Overcome Vaccine Hesitancy: A Systematic Review. Syst. Rev..

[47] Fotiadis K., Dadouli K., Avakian I., Bogogiannidou Z., Mouchtouri V.A., Gogosis K., Speletas M., Koureas M., Lagoudaki E., Kokkini S. (2021). Factors Associated with Healthcare Workers’ (HCWs) Acceptance of COVID-19 Vaccinations and Indications of a Role Model towards Population Vaccinations from a Cross-Sectional Survey in Greece, May 2021. Int. J. Environ. Res. Public Health.

[48] Papagiannis D., Rachiotis G., Malli F., Papathanasiou I.V., Kotsiou O., Fradelos E.C., Giannakopoulos K., Gourgoulianis K.I. (2021). Acceptability of Covid-19 Vaccination among Greek Health Professionals. Vaccines.

[49] Pennisi F., Genovese C., Gianfredi V. (2024). Lessons from the COVID-19 Pandemic: Promoting Vaccination and Public Health Resilience, a Narrative Review. Vaccines.

[50] Rongen A., Robroek S.J.W., Van Lenthe F.J., Burdorf A. (2013). Workplace Health Promotion: A Meta-Analysis of Effectiveness. Am. J. Prev. Med..

[51] Proper K.I., Van Oostrom S.H. (2019). The Effectiveness of Workplace Health Promotion Interventions on Physical and Mental Health Outcomes—A Systematic Review of Reviews. Scand. J. Work Environ. Health.

[52] Joyce S., Modini M., Christensen H., Mykletun A., Bryant R., Mitchell P.B., Harvey S.B. (2016). Workplace Interventions for Common Mental Disorders: A Systematic Meta-Review. Psychol. Med..

[53] Sorensen G., Landsbergis P., Hammer L., Amick B.C., Linnan L., Yancey A., Welch L.S., Goetzel R.Z., Flannery K.M., Pratt C. (2011). Preventing Chronic Disease in the Workplace: A Workshop Report and Recommendations. Am. J. Public Health.

[54] The New European Framework for Action on Integrated Health Services Delivery. https://www.who.int/europe/multi-media/item/the-new-european-framework-for-action-on-integrated-health-services-delivery.

[55] Ammendolia C., Côté P., Cancelliere C., Cassidy J.D., Hartvigsen J., Boyle E., Soklaridis S., Stern P., Amick B. (2016). Healthy and Productive Workers: Using Intervention Mapping to Design a Workplace Health Promotion and Wellness Program to Improve Presenteeism. BMC Public Health.

[56] Poscia A., Moscato U., La Milia D.I., Milovanovic S., Stojanovic J., Borghini A., Collamati A., Ricciardi W., Magnavita N. (2016). Workplace Health Promotion for Older Workers: A Systematic Literature Review. BMC Health Serv. Res..

[57] Bergerman L., Corabian P., Harstall C. (2009). Effectiveness of Organizational Interventions for the Prevention of Workplace Stress. IHE Rep..

[58] Proper K., Van Mechelen W. (2007). Effectiveness and Economic Impact of Worksite Interventions to Promote Physical Activity and Healthy Diet.

[59] Caring for Those Who Care: Guide for the Development and Implementation of Occupational Health and Safety Programmes for Health Workers. https://www.who.int/publications/i/item/9789240040779.

[60] Good Practice in Occupational Health Services: A Contribution to Workplace Health. https://iris.who.int/handle/10665/107448.

[61] Global Strategy on Occupational Health for All: The Way to Health at Work. https://wkc.who.int/resources/publications/i/item/global-strategy-on-occupational-health-for-all-the-way-to-health-at-work.

[62] Buijs P., Gunnyeon B., Van Weel C. (2012). Primary Health Care: What Role for Occupational Health?. Br. J. Gen. Pract..

[63] Safe and Healthy Workplaces in Europe: Where Do We Stand in 2023?|Safety and Health at Work EU-OSHA. https://osha.europa.eu/en/publications/safe-and-healthy-workplaces-europe-where-do-we-stand-2023.

[64] European Agency for Safety & Health at Work—Information, Statistics, Legislation and Risk Assessment Tools. https://osha.europa.eu/en.

[65] Health Services Delivery Programme, Division of Health Systems and Public Health (2016). The European Framework for Action on Integrated Health Services Delivery: An Overview.

[66] Mental Health and Work: Impact, Issues and Good Practices. https://iris.who.int/handle/10665/42346.

[67] Psychosocial Risks in Europe: Prevalence and Strategies for Prevention | European Foundation for the Improvement of Living and Working Conditions. https://www.eurofound.europa.eu/en/publications/2014/psychosocial-risks-europe-prevalence-and-strategies-prevention.

[68] Sun L., Booth A., Sworn K. (2024). Adaptability, Scalability and Sustainability (ASaS) of Complex Health Interventions: A Systematic Review of Theories, Models and Frameworks. Implement Sci..

[69] Anders C., Moorthy P., Svensson L., Müller J., Heinze O., Knaup P., Wallwiener M., Deutsch T.M., Le T.-V., Weinert L. (2024). Usability and User Experience of an MHealth App for Therapy Support of Patients with Breast Cancer: Mixed Methods Study Using Eye Tracking. JMIR Hum. Factors.

[70] Overdijkink S.B., Velu A.V., Rosman A.N., van Beukering M.D.M., Kok M., Steegers-Theunissen R.P.M. (2018). The Usability and Effectiveness of Mobile Health Technology–Based Lifestyle and Medical Intervention Apps Supporting Health Care during Pregnancy: Systematic Review. JMIR Mhealth Uhealth.

[71] Regan C., Von Rosen P., Andermo S., Hagströmer M., Johansson U.B., Rossen J. (2024). The Acceptability, Usability, Engagement and Optimisation of a MHealth Service Promoting Healthy Lifestyle Behaviours: A Mixed Method Feasibility Study. Digit Health.

[72] Rowland S., Ramos A.K., Trinidad N., Quintero S., Johnson Beller R., Struwe L., Pozehl B. (2022). Feasibility, Usability and Acceptability of a MHealth Intervention to Reduce Cardiovascular Risk in Rural Hispanic Adults: Descriptive Study. JMIR Form. Res..

[73] Bergevi J., Andermo S., Woldamanuel Y., Johansson U.B., Hagströmer M., Rossen J. (2022). User Perceptions of EHealth and MHealth Services Promoting Physical Activity and Healthy Diets: Systematic Review. JMIR Hum. Factors.

[74] Spanakis M., Sfakianakis S., Kallergis G., Spanakis E.G., Sakkalis V. (2019). PharmActa: Personalized Pharmaceutical Care EHealth Platform for Patients and Pharmacists. J. Biomed. Inf..

[75] Olawade D.B., Wada O.J., David-Olawade A.C., Kunonga E., Abaire O., Ling J. (2023). Using Artificial Intelligence to Improve Public Health: A Narrative Review. Front. Public Health.

[76] Iribarren S.J., Akande T.O., Kamp K.J., Barry D., Kader Y.G., Suelzer E. (2021). Effectiveness of Mobile Apps to Promote Health and Manage Disease: Systematic Review and Meta-Analysis of Randomized Controlled Trials. JMIR Mhealth Uhealth.

[77] Mezzalira E., Canzan F., Marini G., Longhini J., Leardini C., Saiani L., Ambrosi E. (2024). Introduction of Novel Complex Integrated Care Models Supported by Digital Health Interventions in European Primary Settings: A Scoping Review. Health Policy Technol..

[78] Oh A., Abazeed A., Chambers D.A. (2021). Policy Implementation Science to Advance Population Health: The Potential for Learning Health Policy Systems. Front. Public Health.

[79] Newman M.W. (2022). Value Added? A Pragmatic Analysis of the Routine Use of PHQ-9 and GAD-7 Scales in Primary Care. Gen. Hosp. Psychiatry.

[80] Carroll H.A., Hook K., Perez O.F.R., Denckla C., Vince C.C., Ghebrehiwet S., Ando K., Touma M., Borba C.P.C., Fricchione G.L. (2020). Establishing Reliability and Validity for Mental Health Screening Instruments in Resource-Constrained Settings: Systematic Review of the PHQ-9 and Key Recommendations. Psychiatry Res..

[81] Ford J., Thomas F., Byng R., McCabe R. (2020). Use of the Patient Health Questionnaire (PHQ-9) in Practice: Interactions between Patients and Physicians. Qual. Health Res..

[82] Iles R., Sheppard D.M. (2025). Establishing Key Domains for Measuring Workplace Mental Health: The Indicators of A Thriving Workplace Survey. J. Occup. Rehabil..

[83] Wagner S.L., Koehn C., White M.I., Harder H.G., Schultz I.Z., Williams-Whitt K., Wärje O., Dionne C.E., Koehoorn M., Pasca R. (2016). Mental Health Interventions in the Workplace and Work Outcomes: A Best-Evidence Synthesis of Systematic Reviews. Int. J. Occup. Environ. Med..

[84] Pearl R.L. (2018). Weight Bias and Stigma: Public Health Implications and Structural Solutions. Soc. Issues Policy Rev..

[85] Gray N.S., Davies H., Snowden R.J. (2020). Reducing Stigma and Increasing Workplace Productivity Due to Mental Health Difficulties in a Large Government Organization in the UK: A Protocol for a Randomised Control Treatment Trial (RCT) of a Low Intensity Psychological Intervention and Stigma Reduction Programme for Common Mental Disorder (Prevail). BMC Public Health.

[86] Biswas A., Begum M., Van Eerd D., Johnston H., Smith P.M., Gignac M.A.M. (2022). Integrating Safety and Health Promotion in Workplaces: A Scoping Review of Facilitators, Barriers, and Recommendations. Health Promot. Pr..

[87] Wilcox A., Koontz A. (2022). Workplace Well-Being: Shifting from an Individual to an Organizational Framework. Sociol. Compass.

[88] Guest D.E. (2017). Human Resource Management and Employee Well-Being: Towards a New Analytic Framework. Hum. Resour. Manag. J..

[89] Motalebi G. M., Keshavarz Mohammadi N., Kuhn K., Ramezankhani A., Azari M.R. (2018). How Far Are We from Full Implementation of Health Promoting Workplace Concepts? A Review of Implementation Tools and Frameworks in Workplace Interventions. Health Promot. Int..

[90] Review of Social Determinants and the Health Divide in the WHO European Region. https://www.who.int/publications/i/item/9789289000307.

[91] Andersen J.H., Malmros P., Ebbehoej N.E., Flachs E.M., Bengtsen E., Bonde J.P. (2019). Systematic Literature Review on the Effects of Occupational Safety and Health (OSH) Interventions at the Workplace. Scand. J. Work Environ. Health.

[92] Bondebjerg A., Filges T., Pejtersen J.H., Kildemoes M.W., Burr H., Hasle P., Tompa E., Bengtsen E. (2023). Occupational Health and Safety Regulatory Interventions to Improve the Work Environment: An Evidence and Gap Map of Effectiveness Studies. Campbell Syst. Rev..

[93] Walters D., Johnstone R. (2021). Improving Compliance with Occupational Safety and Health Regulations: An Overarching Review European Risk Observatory Literature Review.

[94] Brenner H., Heisser T., Cardoso R., Hoffmeister M. (2024). Reduction in Colorectal Cancer Incidence by Screening Endoscopy. Nat. Rev. Gastroenterol. Hepatol..

[95] Heisser T., Sergeev D., Hoffmeister M., Brenner H. (2024). Contributions of Early Detection and Cancer Prevention to Colorectal Cancer Mortality Reduction by Screening Colonoscopy: A Validated Modeling Study. Gastrointest. Endosc..

[96] Shattock A.J., Johnson H.C., Sim S.Y., Carter A., Lambach P., Hutubessy R.C.W., Thompson K.M., Badizadegan K., Lambert B., Ferrari M.J. (2024). Contribution of Vaccination to Improved Survival and Health: Modelling 50 Years of the Expanded Programme on Immunization. Lancet.

[97] Braveman P., Gottlieb L. (2014). The Social Determinants of Health: It’s Time to Consider the Causes of the Causes. Public Health Rep..

[98] Verra S.E., Benzerga A., Jiao B., Ruggeri K. (2018). Health Promotion at Work: A Comparison of Policy and Practice Across Europe. Saf. Health Work.

[99] Gagliardi D., Marinaccio A., Valenti A., Iavicoli S. (2012). Occupational Safety and Health in Europe: Lessons from the Past, Challenges and Opportunities for the Future. Ind. Health.

[100] Baicker K., Cutler D., Song Z. (2010). Workplace Wellness Programs Can Generate Savings. Health Aff..

[101] Tsiligianni I., Anastasiou F., Antonopoulou M., Chliveros K., Dimitrakopoulos S., Duijker G., Kounalakis D., Makri K., Petraki C., Prokopiadou D. (2013). Greek Rural Gps’ Opinions on How Financial Crisis Influences Health, Quality of Care and Health Equity. Rural Remote Health.

[102] Carter M.W., Simone P.M., Houry D.E., Reynolds S.L., Patterson S.S., Carlson J.E., Dauphin L.A. (2024). Centers for Disease Control and Prevention’s Public Health Infrastructure Grant: A Better Approach to Empowering More State and Local Decision Making and Strengthening the Public Health Workforce and Infrastructure. J. Public Health Manag. Pr..

[103] Committee on Public Health Strategies to Improve Health (2012). Funding Sources and Structures to Build Public Health.

[104] Spanakis M., Stamou M., Boultadaki S., Liantis E., Lionis C., Marinos G., Mariolis A., Matthaiou A.M., Mihas C., Mouchtouri V. (2025). Enhancing Competencies and Professional Upskilling of Mobile Healthcare Unit Personnel at the Hellenic National Public Health Organization. Healthcare.

[105] Empowerment Through Digital Health. https://www.who.int/europe/initiatives/empowerment-through-digital-health.

[106] Odone A., Buttigieg S., Ricciardi W., Azzopardi-Muscat N., Staines A. (2019). Public Health Digitalization in Europe. Eur. J. Public Health.

[107] Alderwick H., Hutchings A., Briggs A., Mays N. (2021). The Impacts of Collaboration between Local Health Care and Non-Health Care Organizations and Factors Shaping How They Work: A Systematic Review of Reviews. BMC Public Health.

[108] Higgins O., Short B.L., Chalup S.K., Wilson R.L. (2023). Artificial Intelligence (AI) and Machine Learning (ML) Based Decision Support Systems in Mental Health: An Integrative Review. Int. J. Ment. Health Nurs..

[109] Tabrizi J.S., Azami-aghdash S., Gharaee H. (2020). Public-Private Partnership Policy in Primary Health Care: A Scoping Review. J. Prim. Care Community Health.

[110] Awad A., Trenfield S.J., Pollard T.D., Ong J.J., Elbadawi M., McCoubrey L.E., Goyanes A., Gaisford S., Basit A.W. (2021). Connected Healthcare: Improving Patient Care Using Digital Health Technologies. Adv. Drug Deliv. Rev..

[111] Plans-Rubió P. (2022). Strategies to Increase the Percentages of Vaccination Coverage. Vaccines.

[112] WHO Mental Health at Work. https://www.who.int/news-room/fact-sheets/detail/mental-health-at-work.

