# Supplementary material for: Implementing a Mixed Health Service Model as an Informed Modality to Enhance Prevention and Promote Workplace Health in the Greek Regional Public Sector: A Pilot Study in Crete
Source: Healthcare (Basel). 2025 Sep 17;13(18):2337. doi: 10.3390/healthcare13182337 (PMC12469704; doi:10.3390/healthcare13182337)
Supplement: Supplementary file 1 [file healthcare-13-02337-s001.zip › healthcare-3829033-supplementary.pdf]

Table S1. Multiple logistic regression analysis of increased levels of Perceived Stress Scale (PSS-14) and Patient Health Questionnaire-9 (PHQ-9) scales of study's participants, in relation to basic characteristics, health habits and the presence of metabolic syndrome (MetS).

	**Perceived Stress Scale (PSS-14) Score** (high vs. low/moderate)			**Patient Health Questionnaire-9 (PHQ-9) Score** (moderate to severe vs. none-minimal-mild)		
**Factors**	odds ratio, OR	95%CIs	p-value	odds ratio, OR	95%CIs	p-value
**Gender** (females vs. males)	1.79	0.75, 4.25	0.188	1.73	0.43, 7.00	0.441
**Age** (50+ vs. <50yrs)	1.04	0.42, 2.59	0.936	0.54	0.14, 2.12	0.374
**Education level** (per level change: 1. High school, 2. University / Technical School, 3. MSc, 4. PhD)	0.94	0.59, 1.50	0.805	1.49	0.70, 3.17	0.303
**Smoking** (yes vs. no)	0.71	0.27, 1.89	0.496	3.36	0.86, 13.11	0.081
**Alcohol consumption** (yes vs. no)	1.36	0.39, 4.72	0.626	2.48	0.42, 14.76	0.317
**Sleep hours at night** (per hour increase)	0.89	0.64, 1.23	0.468	0.89	0.54, 1.45	0.633
**MetS risk factors** (3+ vs. 0-2 factors)	1.31	0.57, 3.01	0.528	4.11	1.07, 15.81	0.040
R^2 *Negelkerke*		0.029			0.128	

95%CIs, 95% confidence intervals.

Table S2. Hierarchical frequency of 46 recorded intervention types (recommendations/prescription) in the current examination among the 154 participants.

**Rank**	**Proactive action/ recommendation/prescription**	**n**	**%**
1	Recommendation for Tdap vaccination	135	87.7
2*	Recommendation for herpes zoster vaccination (RZV)	18	69.2
3*	Recommendation for PCV20 vaccination	39	60.9
4	Prescription for HbA1c testing	78	50.6
5*	Recommendation for colonoscopy	49	43.8
6	Prescription for kidney function testing	65	42.2
7	Prescription for blood glucose testing	61	39.6
8	Prescription for liver enzyme testing (transaminases)	61	39.6
9	Prescription for complete blood count	58	37.7
10	Prescription for cholesterol/lipid profile testing	58	37.7
11*	Recommendation for weight reduction	48	31.2
12*	Prescription for mammography	24	22.6
13*	Prescription for PSA testing	11	22.4
14	Recommendation for stress management	33	21.4
15*	Recommendation for smoking cessation	28	18.2
16	Portable spirometry on site	25	16.2
17	Recommendation for spirometry at a pulmonology clinic	25	16.2
18	Prescription for bone density scan (DEXA)	24	15.6
19	Recommendation for preventive cardiology evaluation	19	12.3
20	Prescription for vitamin D3 supplement	18	11.7
21*	Recommendation for Pap smear	12	11.2

22	Prescription for lipid-lowering medication	16	10.4
23	Recommendation for HPV DNA test	16	10.4
24	Prescription for ultrasound	14	9.1
25	Recommendation to initiate anxiolytic therapy	13	8.4
26	Prescription for oral glucose tolerance test (OGTT)	12	7.8
27	Recommendation for home blood pressure monitoring	12	7.8
28	Prescription for calcium supplement	11	7.1
29	Recommendation for fecal immunochemical self-test for colorectal cancer	9	5.8
30	Prescription for low-dose chest CT	7	4.5
31	Prescription for iron supplement	5	3.2
32	Prescription for CT scan	4	2.6
33	Recommendation for lipid-lowering diet	4	2.6
34	Referral to a specialist outpatient clinic	3	1.9
35	Prescription for long-term pharmacotherapy	3	1.9
36	Prescription for magnesium supplement	3	1.9
37	Prescription for vitamin B12 supplement	3	1.9
38	Recommendation for influenza vaccination	3	1.9
39	Prescription for chest X-ray	2	1.3
40	Prescription for hormonal testing	2	1.3
41	Prescription for chronic pain medication	2	1.3
42	Prescription for breast ultrasound	1	0.9
43	Prescription for lower limb venous triplex ultrasound	1	0.6
44	Recommendation for blood donation	1	0.6
45	Recommendation for liver evaluation	1	0.6
46	Recommendation for neurology evaluation	1	0.6

* *The percentages are based on the number of individuals in each subgroup estimated according to their demographics or clinical characteristics*

Data collection procedures

1) Blood pressure:

The equipment used for the measurement of blood pressure was an A&D UA-767S-W Afib Digital Upper Arm Blood Pressure Monitor (https://www.aandd.jp/products/manual/medical/ua767sw_en.pdf), which has received the highest clinical rating based on the British & Irish Hypertension Society (BIHS) protocol. The 2021 European Society of Hypertension guidelines recommend the use of validated automated electronic (oscillometric) upper-arm blood pressure monitors for clinical practice, noting that such devices are now increasingly preferred for office measurements due to their reliability and ability to provide standardized, repeat readings. Blood pressure measurements were taken after the initial history-taking and as the initial step in the physical examination, once the patient had time to sit quietly. Ideally, the patient rested for at least five minutes in a seated position with their back support, feet flat on the floor, and arm positioned at heart level. To avoid artificially elevated readings, the patient was questioned regarding caffeine consumption, smoking, exercise, and alcohol consumption 30 minutes prior to the interview. This preparation ensured that by the time the blood pressure measurement was taken - typically about midway through the consultation, following discussion and before further physical assessments - the result reflected the patient’s true state of resting.

Reference: https://pubmed.ncbi.nlm.nih.gov/33710173/

2) Waist circumference

Waist circumference was measured according to standard practice using a GIMA 27343 body measuring tape. Participants stood upright with arms slightly away from the body while a trained member of the interdisciplinary team positioned the tape at the correct anatomical level. Specifically, the upper hip bone and the top of the right iliac crest were located, and the tape was placed in a horizontal plane around the abdomen at the level of the iliac crest. The tape was held snug but not compressing the skin, parallel to the floor, and the measurement was taken at the end of a normal expiration.

3) Oxygen saturation

The device used for the measurement of the patients' oxygen saturation was the Beurer PO35 Finger Oximeter (https://www.beurer.com/global/p/45431/)
